# Learning from video tutorials: mental focusing and rehearsal are just as effective as written focusing

**DOI:** 10.3389/fpsyg.2026.1768486

**Published:** 2026-06-04

**Authors:** Simon A. Schriek, Kirsten Berthold, Markus H. Hefter

**Affiliations:** Department of Psychology, Bielefeld University, Bielefeld, Germany

**Keywords:** cognitive load, focused processing, mental rehearsal, prompts, self-regulated learning, video tutorials

## Abstract

**Introduction:**

Learning with video tutorials has grown in popularity during the last years. Taking recourse to video tutorials enables learners to learn at their own convenience. Yet without the presence of instructors, learners need to self-regulate their learning process which novices in particular find overwhelming. Focus prompts can support learners to do this. While answers to such prompts are usually entered via a keyboard, the growing use of immersive technologies demands for prompts that do not rely on text entries.

**Methods:**

In this study, we compared a written focus condition (written answers to prompts) with a mental focus condition (creating a mental list), a mental rehearsal condition (performing actions before the inner eye) and a control condition without instructional support in a sample of 117 university students. We used industrial original video tutorials in our study.

**Results:**

We did not find any differences between the various conditions with regard to learning engagement, learning outcomes, and instructional efficiency (i.e., relative efficiency combining learning outcomes and cognitive load) but differences in learning time: participants in the written focus condition needed more time to respond to the prompts.

**Discussion:**

We attribute the lack differences in learning engagement and outcomes to the well-designed learning environment and the highly controlled laboratory setting. Our findings show that it is possible to learn with video tutorials without responding to prompts in written form and implicate that learners' time and cognitive resources should be saved when the learning material is sufficient.

## 1 Introduction

During the past few years, learning with instructional videos has grown in popularity (Giannakos, 2013; Sablić et al., 2021) and video tutorials especially have become an integral part of modern education. Video tutorials typically show an expert demonstrating several working steps while giving step-by-step instructions (van der Meij and van der Meij, 2015). A prime example for the rise in video tutorials is the platform YouTube, which is widely used for learning with videos. This platform hosts a plethora of do-it-yourself videos, offering step-by-step instructions on a wide array of topics from crafting tutorials to plumbing (Hefter, 2024) to instructions for beauty procedures like applying botox (Castillo-Abdul et al., 2021). One reason for the popularity of such how-to-videos is their permanent accessibility. Learners can access learning material at their own convenience at any place or time, offering the possibility of learning in absence of teachers and supervisors (Anggraeni and Theedens, 2024; Hanim et al., 2023).

With no teacher present, learners must control and regulate their learning behavior themselves. Self-regulated learning therefore plays a key role in e-learning (Yu, 2022). It entails learners' ability to independently manage their learning process by actively monitoring, regulating, and controlling, inter alia, cognitive aspects of learning (Zimmermann, 2002). When learning with video tutorials, learners face the challenge of following the working steps on display and their explanations. Cognitive learning strategies help learners do this. These strategies aim at the processing of information learners are provided with. The effectivity of cognitive learning strategies can be explained by the Cognitive Theory of Multimedia Learning (CTML; Mayer, 2021). Learners need to engage in active cognitive processes to learn. The CTML describes three cognitive processes: the selection of key information (Selecting), giving this information a coherent structure (Organizing), and creating links to prior knowledge (Integrating). While the working steps are the key information shown in video tutorials (Schriek et al., 2025), learners are often also given additional background information (Chandra et al., 2023). In order to learn, they need to identify the working steps and distinguish them from one another, they need to create a coherent mental representation of the working steps, and make connections to the knowledge they already possess.

Learners need to allocate cognitive resources to execute these cognitive processes. Because they are limited, allocating such resources is difficult (Mayer, 2021). This idea is highlighted by the Cognitive Load Theory (CTL; Sweller et al., 2011). The CTL distinguishes between intrinsic load (i.e., the difficulty of learning material), extraneous load (i.e., the way information is presented), and germane load (i.e., cognitive resources devoted to processing the presented information). Against the background of the CTML and the CLT, Mayer and Moreno (2003) postulate three different demands for cognitive processes when learning with multimedia: essential processing (i.e., making sense of the material), incidental processing (i.e., processing non-essential information), and representational holding (i.e., holding representations in working memory). When creating learning contents, one should attempt to maximize learners' resources spent on essential processing (e.g., by helping learners to engage in Selecting, Organizing or Integrating), while minimizing incidental processing and representational holding. In other words, learners should be able to concentrate on making sense of the presented information instead of processing non-essential aspects (e.g., seductive details; Garner et al., 1989) or spending a lot of working memory capacities on keeping presented information in mind. One way to establish the cognitive resources learners have allocated is by asking them, how much mental effort they invested while learning (Paas and van Merriënboer, 1993). This question is important because it has further theoretical and practical implications. The assessment of learning outcomes (e.g., posttests on declarative knowledge) helps to answer the question, whether participants in an experimental condition learned more than those in a control condition (i.e., effectivity). The additional assessment of mental effort helps determine how many cognitive resources had to be allocated to learn (i.e., efficiency). High effort is not necessarily the same as learning effectively, and high learning outcomes do not necessarily imply efficient learning (Bruin et al., 2020). An elegant way to account for questions of efficiency is to interrelate learning outcomes and mental effort (Hoffmann and Schraw, 2010; Paas and van Merriënboer, 1993). Learning environments and instructional design should pursue learning that is both effective and efficient.

When resorting to video tutorials, learners can find effective and efficient learning to be particularly difficult. First of all, all information presented (pictorial and verbal) is transient for learners (transient information effect; Singh et al., 2012). In contrast to written information like texts, the information learners receive via videos is not permanent and needs to be held active in working memory. When watching a video tutorial on operating an industrial drilling machine for example, learners must keep both the verbal instructions and their demonstration in mind in addition to any further information (e.g., safety precautions or technical terms) while also having to comprehend the presented information. This is particularly challenging for novice learners, as they have high cognitive demands for essential processing (i.e., understanding the video contents) as well as high demands for representational holding, potentially leading to cognitive overload. In addition, learning with videos can create a passive learning situation (Erickson et al., 2020). Such a passive learning situation is characterized by learners receiving information (e.g., via video contents) without engaging with the presented information. As a result, learners can be under the illusion of understanding, which is a misjudgment of their own comprehension (e.g., Kardas and O'Brien, 2018). When learning with video tutorials for example, one might watch an expert performing several working steps on an industrial machine without any engagement in grasping the most important information displayed in the tutorial (i.e., passive learning). In consequence, one might think to be able to perform or name these working steps without being able to do so (i.e., illusion of understanding). To overcome passiveness and the potential illusion of understanding, learners can apply cognitive learning strategies (e.g., creating a list of the most important working steps of a video tutorial). However, learners might fail to apply cognitive learning strategies without an instruction telling them to do so (Bisra et al., 2018). This becomes especially apparent in novices (i.e., learners without prior domain knowledge): Prior knowledge plays an integral part in the effective utilization of cognitive learning strategies and serves as a foundational component for learners to engage with material (Fogel, 2022). From a CLT perspective, managing learners' limited cognitive resources is crucial for learning (Sweller et al., 2011). Novice learners in particular may struggle to cope with too much information, leading to cognitive overload and negatively affecting their comprehension and engagement (Sweller et al., 2011). For example, novice learners might be overwhelmed by selecting key information because such an assessment (i.e., determining what the most important information is) requires prior knowledge (Roelle et al., 2015). As a consequence, learners need instructional support beyond video tutorials to help them focus on key aspects of learning contents and to ensure learning. Accordingly, the so-called focused processing stance (Renkl and Atkinson, 2007; Renkl, 2015) builds on the idea of active processing: learners need to be enabled to focus on key components rather than to merely process learning material as a whole. One way to do this is by prompting learners to interact with the most relevant information in the learning material. Schriek et al. (2025) found that retrospective focus prompts were effective when learning with technical video tutorials. Giving learners the task to list the most important working steps shown in the video tutorials led to better learning outcomes in comparison to a control condition without instructional support. Focus prompts support learners in different ways. On the one hand, they draw their attention toward key information shown in the video tutorials and on the other hand, they evoke a generative learning activity (Fiorella and Mayer, 2016, 2021). Learners are encouraged to summarize what they have seen and heard in their own words, hence creating a coherent mental representation of learning contents.

Yet there are also drawbacks to such prompts. First, they take time to answer (Schriek et al., 2025). Second, a keyboard is needed to type in answers. The use of immersive technologies (e.g.; augmented reality) is growing in educational contexts (Bödding et al., 2025). These technologies usually lack an (analog) keyboard and pose challenges for extended text input. This triggers the question of instructional support that does not depend on text entries. One simple way to overcome this challenge is by prompting learners to create a list of the most important working steps in their mind instead of writing them down. There are open questions as to whether learners engage with learning materials without needing to type in answers. From a cognitive load point of view, creating a list in one's mind might be more demanding for learners because they have to keep all the working steps in their working memory. Given the limited capacity of working memory, this could lead to cognitive overload and hinder effective learning (Mayer, 2021). An alternative is the task to mentally rehearse the working steps learners have seen. Mental rehearsal instructs learners to repeat the working steps shown before their inner eye. Typically, such tasks aim at acquiring skills (i.e., procedural knowledge), and there is various research on the beneficial effects of mental rehearsal on procedural knowledge (e.g., Trabelsi et al., 2022). The Adaptive Control of Thought theory (Anderson, 1982) describes declarative knowledge as the first step in skill acquisition. Before being able to correctly execute working steps, learners need first to know what the correct working steps are. Mental rehearsal is a supplement to practical training and an intermediate step on the path from novice to expert (Sanders et al., 2007). As mental rehearsal engages similar neural circuits involved in an actual motor performance (e.g., Cisek and Kalaska, 2004), it helps to learn the execution of a certain task. Hence, it aims at acquiring procedural knowledge. The act of naming a working step (i.e., declarative knowledge) entails different cognitive processes than implementing the knowledge of a working step (i.e., procedural knowledge; Morehead et al., 2011). Because declarative knowledge is needed to acquire procedural knowledge, and novice learners lack this foundation of declarative knowledge, there are open questions whether learners without prior knowledge actually benefit of mental rehearsal with regard to the acquisition of declarative knowledge.

As described above, the aim of prompts is to enable learners to interact with learning content. There are different ways to determine whether such an interaction has taken place. Typically, answers to prompts are analyzed to make statements about learning engagement. If an answer to a prompt includes contents of learning material, this indicates that learners have elaborated the information they had just seen, read, or heard. For example, to analyze learning engagement, Schriek et al. (2025) looked at the number of correct working steps listed in the reaction to their prompts. However, for such an analysis, the learner needs to document such answers to prompts, either in written form (e.g., Schriek et al., 2025) or orally (e.g., van den Broek et al., 2025). When instructing learners to create a list in their mind or to mentally rehearse contents of video tutorials, there are no answers to prompts that can be analyzed. To overcome this challenge, learners can be asked *post-hoc* to self-assess their learning engagement (Daumiller et al., 2020; Hefter and Nitsch, 2024).

## 2 Research questions

Against this background, we investigated the primary research questions below:

Do the different prompt types lead to…

1) … differences in learning engagement?
2) …differences in learning outcomes (i.e., free recall of working steps)?
3) … differences in cognitive load?

On a site note, we also investigated the secondary research question below:

4) Do the different prompt types lead to differences in learning time?

## 3 Method

### 3.1 Sample and design

We conducted an a priori power analysis using the software G^*^Power 3.1.9.7 (Faul et al., 2007) to determine the required sample size for an F test (fixed effects, omnibus, one-way). We based our estimated effect size on a study by Schriek et al. (2025) who worked with similar videos and prompts. The parameters we used to determine the required sample size were: *f* = 0.30, α = 0.05, and a power of 0.80. The sample should contain 128 participants according to this analysis. One-hundred and seventeen university students (74 female, 43 male; *M*_*Age*_ = 24.68, SD_*Age*_ = 5.24) participated in this study. All participants were randomly assigned to one of the four experimental conditions featuring different prompt types: written focus (*n* = 30), mental focus (*n* = 29), mental rehearsal (*n* = 31), no prompt (*n* = 27). Ninety-four students participated in the delayed posttest 2 to 3 weeks after the initial experiment (~ 20% dropouts). More specifically, sample sizes at the delayed posttest were as follows: written focus (*n*_delayed_ = 20), mental focus (*n*_delayed_ = 23), mental rehearsal (*n*_delayed_ = 28), no prompt (*n*_delayed_ = 24). We used a chi-square test to compare the experimental condition and participation in the delayed posttest. The results showed no significant relationship between experimental condition and participation in the delayed posttest; $\chi_{\left(3\right)}^{2}$ = 6,95, *p* = 0.073.

### 3.2 Materials

The topic was introduced to participants via text- and picture-based explanations on the learning goals, the overall functionality of an industrial drilling machine and assisting tools as well as an overview of the working steps to be displayed in the video tutorials. After this introduction to the topic, participants watched three video tutorials on the handling of an industrial drilling machine. The videos displayed an expert from the point-of-view perspective demonstrating how to put a drilling machine into operation, how workpieces are inserted correctly, and how to drill different kinds of holes. The videos lasted 152 to 216 s. We used the same introduction and video tutorials as Schriek et al. (2025). After each video, participants were given the prompt to either type a list of the main working steps shown in the previous video into a text box (written focus condition), to create a list of the main working steps in their mind (mental focus condition) or to imagine carrying out the working steps themselves (mental rehearsal condition). Participants in the control condition received no instructional support. Between the videos, there was an intermediate screen informing participants that the next video tutorial would start on the next page. This created a short break between the videos. Table 1 shows the exact wording of the prompts.

**Table 1:** Conditions and prompt types.

Experimental condition	Prompt
Written focus	“What were the main working steps shown in the last video? Please create a list in this text field.”
Mental focus	“What were the main working steps shown in the last video? Please create a list in your mind.”
Mental rehearsal	“Please execute the working steps you have just seen before your inner eye”

All prompts are translated from German.

### 3.3 Measures

#### 3.3.1 Prior knowledge

We assessed prior knowledge via a single open question that addressed the content of the topic's introduction that was posed before the video tutorials were shown. We rated participants' answers to this question on a scale from 0 to 4 with each point representing information from the introduction. We used the same question and the same rating system as Schriek et al. (2025). Both the first author and a student research assistant rated these answers from 38 participants, resulting in an ICC of 0.91. The research assistant rated the remaining data.

#### 3.3.2 Learning time

We tracked how much time it took participants to read the introduction into the topic, to watch the three video tutorials and to react to our prompts. Participants had unlimited time to do this.

#### 3.3.3 Prompt score

We rated the participants' answers in the written focus condition via a rating system that gave one point for each correct working step mentioned in the answers to the prompts. This rating system was closely associated with the video tutorials' content and resulted in a scale from 0 (no working steps mentioned) to 6 (all working steps mentioned). The three prompt answers were rated independently and the mean of the three prompt ratings yielded the prompt score. The first author and a student research assistants rated the answers from 6 participants (20% of the data), resulting in an ICC of 0.94. The student research assistant rated the remaining data.

#### 3.3.4 Learning engagement

We used five items to assess the multi-faceted nature of learning engagement, that have been previously used by Daumiller et al. (2020). The items included statements on learning effort (“I made a lot of effort to understand everything”), learning intensity (“I dealt with the learning content intensely”), elaboration (“I tried to find connections between the current learning topic and what I already know”), implementation (“I have thought about concrete situations in which I can implement what I have just learned”) and persistence (I worked persistently and in a focused manner”). Participants answered these items on scale from 1 (*strongly disagree*) to 8 (*strongly agree*). The internal consistency was α = 0.57.

#### 3.3.5 Cognitive load

We followed Grund et al. (2024) recommendation to use difficulty as a measure of cognitive load and assessed it via a single item that referred to difficulty in style of the Paas item (Paas, 1992). The item's exact wording was “How difficult did you find it to understand the content shown?”. Participants rated this item on a scale from 1 (*lowest*) to 9 (*highest*).

#### 3.3.6 Learning outcomes

We assessed learning outcomes via a free recall task of the working steps displayed in the video tutorials. Participants were tasked with listing all working steps they remembered from the videos. We used the same rating systems described above for the prompt score, resulting in a joint rating scale comprising all the working steps in all tutorials. We used the same question and the same rating system as Schriek et al. (2025). The first author and a student research assistant rated ~20% of the data (32 participants), resulting in an ICC of 0.97. The research assistant rated the remaining data.

#### 3.3.7 Instructional efficiency

We calculated the efficiency of our prompts and applied Paas and van Merriënboer's (1993) formula to do so. The formula subtracts the z-standardized value of performance data from the z-standardized value of cognitive load and divides the difference by the square root of 2.

### 3.4 Procedure

**Figure 1:**
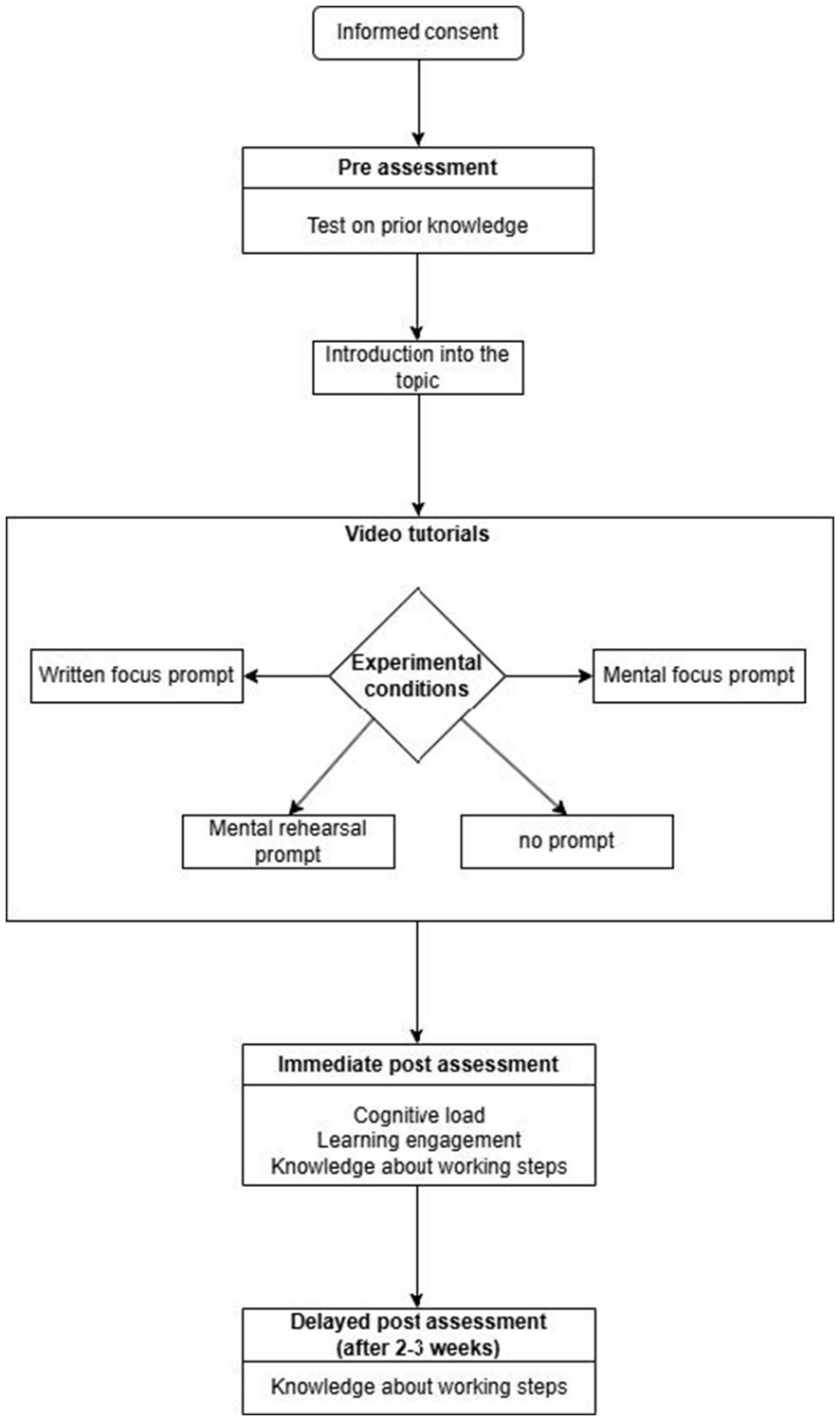
Study's design and procedure.

The experiment took place in a university laboratory, and student research assistants supervised the entire experiment. Participants worked on our PCs and were given noise-canceling headphones. The student research assistants instructed participants that they were only allowed to take notes within the digital learning environment when instructed to do so. The student research assistants monitored the entire experiment. Before watching the video tutorials, participants were given a short test on prior knowledge and an introduction into the topic. The introduction could be reread if participants wished to do so. Participants then watched the three video tutorials and had the option to rewind and pause the videos. After each video tutorial, participants were given a prompt according to their experimental condition. Once a prompt was given, participants could not go back to the respective video tutorial. Participants in the control condition were given no prompt and were directly presented the intermediate screen after having watched the video tutorial. This screen informed participants that the next video tutorial would start on the next page. After having watched the video tutorials and reacted to the prompts, participants took part in an immediate posttest consisting of questions on learning engagement, as well as a free recall of the working steps shown in the tutorials. Participants were then invited to take part in the delayed posttest after 2 weeks. The delayed posttest was online, not in our university laboratory. Participants were given 1 week to participate in the delayed posttest. Figure 1 shows the study's design and procedure in a flowchart.

## 4 Results

We applied ANOVAs for all four research questions and report $\eta_{\text{p}}^{2}$ as effect size for *F* tests, qualifying as small ($\eta_{\text{p}}^{2}$ <0.06), medium ($\eta_{\text{p}}^{2}$ between 0.06 and 0.13) or large ($\eta_{\text{p}}^{2}$ > 0.13, Cohen, 1988). We applied 0.05 as the alpha level and Bonferroni-Holmes adjustments for all *post-hoc* comparisons and report *d* as effect size for *t* tests, qualifying as small (|*d*| between 0.20 and 0.49), medium (|*d*| between 0.50 and 0.79) or large (|*d*| > 0.80; Cohen, 1988). For correlation analysis, we report *r* as effect size, qualifying as small (|*r*| <0.30), medium (|*r*| between.30 and.50) or large (|*r*| >0.50). Table 2 shows means and standard deviations for all measures. Table 3 shows Pearson correlations for all measures.

**Table 2:** Means with standard deviations in parentheses for all measures.

Measure	Written focus	Mental focus	Mental rehearsal	No prompt	Overall
Prior knowledge	0.07 (0.25)	0.28 (0.53)	0.26 (0.51)	0.19 (0.48)	0.20 (0.46)
Learning time^a^	25.46 (5.08)	17.85 (4.03)	17.00 (3.33)	17.19 (4.08)	19.42 (5.45)
Prompt score^b^	4.33 (0.73)	—	—	—	4.33 (0.73)
Learning engagement^c^	5.01 (1.39)	5.14 (1.09)	5.53 (0.92)	5.42 (1.19)	5.27 (1.16)
Cognitive load^d^	3.53 (2.13)	3.07 (1.73)	2.90 (1.83)	3.07 (2.00)	3.15 (1.92)
Learning outcomes^e^					
Immediate posttest	7.83 (3.21)	9.00 (3.86)	8.68 (3.71)	8.41 (3.92)	8.48 (3.65)
Delayed posttest	7.05 (2.65)	6.70 (4.01)	7.14 (2.51)	6.67 (2.76)	6.90 (2.98)
Instructional efficiency^f^					
Immediate posttest	−0.27 (1.15)	0.13 (1.18)	0.13 (1.09)	0.01 (1.14)	0.00 (1.14)
Delayed posttest	−0.21 (1.23)	−0.01 (1.12)	0.21 (1.04)	0.11 (1.08)	0.05 (1.10)

^a^in minutes.

^b^Scale from 0 to 6.

^c^Scale from 1 to 8.

^d^Scale from 1 to 9.

^e^Scale from 0 to 17.

^f^Difference the standardized value of cognitive load and standardized value of learning outcomes divided by the square root of 2.

**Table 3:** Pearson correlations for all measures.

Measure	Correlations								
	(1)	(2)	(3)	(4)	(5)	(6)	(7)	(8)	(9)
(1) Prior knowledge								
(2) Learning time	−0.13							
(3) Prompt score	0.19	**0.46**							
(4) Learning engagement	0.15	−0.11	**0.39**						
(5) Cognitive load	−0.15	0.13	−0.41	−0.14					
(6) Learning outcomes (immediate)	0.14	0.13	**0.66**	0.10	–**0.29**				
(7) Learning outcomes (delayed)	**0.25**	0.18	**0.57**	0.11	–**0.29**	**0.51**			
(8) Instructional efficiency (immediate)	**0.18**	0.00	**0.64**	0.15	–**0.80**	**0.80**	**0.51**		
(9) Instructional efficiency (delayed)	**0.24**	0.01	**0.53**	0.17	–**0.79**	**0.49**	**0.82**	**0.80**	

Bold correlations: *p* < 0.05 (two-sided).

### 4.1 Preliminary analysis

There were no statistically significant differences between the experimental conditions regarding prior knowledge, *F*_(3, 113)_ = 1.29, $\eta_{\text{p}}^{2}$ = 0.03, *p* = 0.282.

### 4.2 Learning engagement

#### 4.2.1 Relationship between prompt score and learning engagement

We correlated the prompt score (i.e., answers to the prompts in the written focus condition) with the self-reported learning engagement. The correlation was significant, *r* = 0.39 (medium effect), *p* = 0.032.

#### 4.2.2 Effect of prompt type on learning engagement

An ANOVA showed no effect of prompt type on learning engagement, *F*_(3, 113)_ = 1.31, *p* = 0.276, $\eta_{\text{p}}^{2}$ = 0.03.

### 4.3 Effect of prompt type on cognitive load

An ANOVA showed no effect of prompt type on cognitive load, *F*_(3, 113)_ = 0.60, *p* = 0.619, $\eta_{\text{p}}^{2}=$ 0.02.

### 4.4 Effect of prompt type on learning outcomes

We conducted two separate ANOVAs for the immediate and the delayed posttest. For the immediate posttest, the ANOVA showed no effect of prompt type on learning outcomes, *F*_(3, 113)_ = 0.54, *p* = 0.659, $\eta_{\text{p}}^{2}$ = 0.01. For the delayed posttest, the ANOVA also showed no effect of prompt type on learning outcomes, *F*_(3, 91)_ = 0.16, *p* = 0.923, $\eta_{\text{p}}^{2}$ = 0.01.

### 4.5 Effect of prompt type on instructional efficiency

Again, we conducted two separate ANOVAS for the immediate and the delayed posttest. There were no effects for instructional efficiency for the immediate posttest, *F*_(3, 113)_ = 0.81, *p* = 0.492, $\eta_{\text{p}}^{2}$ = 0.02, and also no effects for instructional efficiency for the delayed posttest, *F*_(3, 91)_ = 0.57, *p* = 0.639, $\eta_{\text{p}}^{2}$ = 0.02.

### 4.6 Effect of prompt type on learning time

An ANOVA showed a statistically significant effect of prompt type on learning time, *F*_(3, 113)_ = 28.37, *p* < 0.001, $\eta_{\text{p}}^{2}$ = 0.43 (large effect). *Post-hoc* tests revealed longer learning time in the written focus condition compared to the mental focus condition, *t*_(58)_ = −7.01, *p* < 0.001, *d* = −1.83 (large effect), compared to the mental rehearsal condition, *t*_(60)_ = −7.92, *p* < 0.001, *d* = −2.03 (large effect), and compared to the control condition, *t*_(56)_ = −7.48, *p* < 0.001, *d* = −1.98 (large effect).

## 5 Discussion

Our study results showed no statistically significant differences in learning engagement or learning outcomes between the different instructional conditions. Whether participants were prompted to type a list of the main working steps, to mentally list them, to imagine performing the working steps or not instructed at all: their reported engagement levels and their performance in a free recall task (listing all working steps they remembered) remained similar. Mayer and Moreno (2003) distinguish between three demands for learners' cognitive resources when learning with multimedia materials: essential processing, incidental processing, and representational holding. These demands help explain the present study's null findings.

Essential processing describes mental processes aiming to make sense of the learning contents, which includes the three processes postulated by the CTML Selecting, Organizing, and Integrating. The aim of our prompts was to support learners at selecting the most important information shown in the videos (i.e., the working steps) by explicitly telling participants to concentrate on the working steps they had just seen and to either write them in a text field (i.e. written focus condition), list them up in their mind (i.e., mental focus condition) or to execute the working steps before their inner eye (i.e., mental rehearsal condition). Taking a descriptive look at our data, the prompt score (i.e., the number of correct working steps typed in the text field) in the written focus condition suggests that participants in that experimental group engaged in selecting the most important information: in the mean, they named 4.33 working steps out of 6 per video tutorial (about 72%), indicating that they indeed focused on the working steps displayed. We have no answers to the prompts for the other three conditions because they either were given no prompt (no prompt condition) or had to react to the prompt in their mind without externalizing it (mental focus condition and mental rehearsal condition). We asked all participants to self-assess their learning engagement via our learning engagement scale. As the prompt score in the written focus condition indicates that participants focused on the working steps in reaction to our prompts and the self-assessed learning engagement was comparable, it is likely that all participants (regardless of their experimental condition) engaged in meaningful learning processes. However, we do not see statistically significant differences between the experimental conditions with regard to learning engagement and we cannot say the extent to which our prompts evoked executing these processes or whether participants applied different learning strategies when watching the video tutorials. It is important to note that our sample consisted of university students who usually have more experience in applying different learning strategies and in self-regulated learning. Research has indicated that students engage in self-regulated learning when they possess high self-efficacy beliefs, which significantly influence their ability to set goals, monitor performance, and evaluate their learning process (Lin et al., 2022). This heightened engagement typically coincides with key academic transitions, such as moving from high school to higher education, where the demand for independent learning is more intense (Lin et al., 2022). Students who know how to monitor their learning process and apply different learning strategies may not need instructional support to learn effectively. This might also be an explanation why we did not find the same effects as Schriek et al. (2025), who found that focusing retrospectively on key information in video tutorials was beneficial in a sample of high school students. In our sample of higher-performing university students, obviously prompting did not result in differences in learning outcomes.

Another demand according to Mayer and Moreno (2003) is incidental processing which refers to the cognitive resources used to process non-essential aspects of learning materials. We applied established multimedia learning principles to keep incidental processing at a minimum. First, the intervention yielded precise pre-training (e.g., Mayer et al., 2002) to introduce learners to the topic by explaining technical terms and explaining how an industrial machine functions. This pre-training likely gave learners a sufficient amount of prior knowledge to make sense of the video contents without further instructional support. In addition, it probably reduced the learners' incidental processing, as they were already familiar with technical terms and the functionality of an industrial drilling machine. Second, the content of the video tutorials was based on a thorough task analysis and scripted beforehand to ensure clarity and consistency in both the visuals and narration. Consequently, the video contents were already condensed to contain the most essential information, which made it obvious to the learners, as to what they needed to focus on, even without a particular instruction to do so.

The third demand according to Mayer and Moreno (2003) is representational holding, which describes cognitive resources that need to be spent to hold information active in working memory. On the one hand, it seems feasible to assume that participants in the written focus condition had to spend fewer resources on representational holding compared to the other conditions, as they were in the only condition that enabled them to externalize working steps by typing them into a text field in response to our prompts. Once a working step had been written down, it no longer had to be kept active in working memory and cognitive resources could be used to process the next working step. The other three conditions excluded note-takings in any form, so participants were likely to have spent more resources on representational holding. Participants in the mental focus condition had to keep all the working steps in their mind and participants in the mental rehearsal condition had to perform the task of operating the drilling machine in their mind (i.e., holding the execution of the working steps active in their working memory). However, we do not see differences in cognitive load between the different experimental conditions. One explanation for this lies in our application of the segmenting principle: we had divided the videos into suitable content segments (Mayer and Moreno, 2003). This division narrowed down the working steps per video so that the amount of information to be held active in working memory was limited and could be processed without cognitive overload, regardless of the prompt condition. Accordingly, there was no incremental benefit of being able to externalize working steps for participants in the written focus condition. As video tutorials are often not segmented, being able to write down working steps might be more important in settings with higher external validity (e.g., learning with longer non-segmented YouTube tutorials). One of the obstacles when learning with videos is that the information is transient (Ayres and Paas, 2007; Castro-Alonso et al., 2021). This obstacle is greater, the longer the video and the more information needs to be held active in working memory. In less controlled settings learners face the challenge of keeping a lot of information active in their working memory at the same time, risking a potential cognitive overload. We avoided such overload by segmenting the video tutorials into content-driven segments.

Our study had many similarities with the one conducted by Schriek et al. (2025), including two similar prompt conditions (i.e., written focus condition and no prompt condition), the same videos and the same posttest measures. These similarities allow us to descriptively compare the concrete results of the two identical prompt conditions between the two studies. Interestingly, our learning outcomes are higher than in Schriek et al. (2025) study: participants in our study were able to name up to twice as many working steps in posttests. Although such a direct comparison should be treated cautiously, it indicates two things: on the one hand, it shows that regardless of their experimental condition, participants in our study learned some of the working steps displayed in the videos as they were able to name up to half of them in a posttest, which is a higher amount compared to the working steps participants could name in a comparable study. Additionally, it does point out that there were factors in our study that influenced participants learning outcomes beyond our learning environment, including the introduction, the videos and our prompts. The most important differences between the current study and the one conducted by Schriek and colleagues are the composition of the sample and the environmental circumstances. Our sample consisted of university students who usually have broader experience applying different learning strategies and self-regulated learning (Lin et al., 2022). With regard to environmental circumstances, our study took place in a highly controlled environment: we conducted the experiment in a university lab and participants wore noise-canceling headphones, providing them with an isolated and distraction-free setting and allowing them to fully concentrate on the video tutorials. In addition, trained student assistants were present throughout the experiment and could give immediate support in case of technical issues, further reducing disruptions from learning. Previous research showed that in asynchronous learning settings, without the presence of instructors, instructional support becomes more important for engaging in learning processes inter alia due to the distractions of smartphones (Hefter, 2025). Furthermore, there is a negative association between the number of interruptions during learning and learning outcomes (Hefter et al., 2023). In an unsupervised setting, smartphones or other devices can also be a source of off-task behavior (Burak, 2012; Hefter et al., 2023; Ochs et al., 2024). The study of Schriek et al. (2025) was conducted in high school classrooms. It is possible that the added value of prompts when learning with video tutorials will only become apparent in a setting with more distractions, in which learners need support to sustain or regain focus on key aspects of learning contents. In a sample likely to have knowledge about the application of learning strategies and in a distraction-free learning context, prompting learners to engage with the learning material (in written form or mentally) obviously did not have a significant increment. These observations are of importance in the context of the so-called assistance dilemma (Koedinger and Aleven, 2007) which refers to the difficulty of finding the right balance between providing assistance to learners and giving them autonomy over their learning process. The expertise reversal effect (Fyfe and Rittle-Johnson, 2016; Kalyuga, 2007, 2021) is closely related to the assistance dilemma: novices benefit from more explicit instruction, while advanced learners may find such instructions redundant (Rey and Buchwald, 2011). Hence, instructions should take prior knowledge into account and be different depending on learners' prior knowledge. The comparison of our findings with those of Schriek et al. (2025) indicates that this assistance dilemma might not only exist with regard to prior domain knowledge but also concerning sample characteristics and the learning context. For learner-tailored instructional support, it is important to also consider how undisturbed they can learn (e.g., an isolated laboratory vs. learning in a classroom) and how much knowledge they possess on applying learning strategies on their own (e.g., high school students vs. university students).

One of our goals was to test instructions not dependent on text entries via a keyboard. Our findings imply that text entries are not necessary to learn, as long as the learning environment takes human information processing into account (Mayer and Fiorella, 2021). In other words, when learning with well-designed and segmented video tutorials in a non-distractive setting, there is no need for text entries to grasp the most important working steps displayed. From a researcher's point of view, the absence of text entries comes along with the question of how to assess learners' learning processes or engagement. We correlated the prompt score (i.e., the rated answers to the prompts in the written focus condition) with the self-assessed learning engagement and could see a significant positive relationship between these two. This indicates that it is possible to assess learning engagement without taking recourse to written or recorded answers. For learning environments where text input is not feasible, these self-assessments could give researchers the opportunity to make assumptions about learning engagement.

On a side note, we were also interested in differences in learning time between our experimental conditions. Our results show that it takes more time to type in a list of the working steps than to create a list in the mind or to replicate the demonstrated working steps before one's inner eye. In absence of statistically significant differences in learning outcomes, reacting to a prompt in mind or not having to react to a prompt at all is apparently more time-efficient than answering a prompt in written form. From an efficiency perspective, one could also look at the standardized difference between performance and effort (e.g., Paas and van Merriënboer, 1993; see also Hoffmann and Schraw, 2010). Although such efficiency indices are no absolute indicators for learning, they give us an idea about the relation between invested effort and learning outcomes. When considering knowledge about working steps as performance and cognitive load as effort, the written focus group even had a negative efficiency value, indicating less efficiency than the other conditions. In other words, participants in the written focus condition invested more cognitive resources without any return on their investment in form of higher learning outcomes. Although the differences in instructional efficiency are not statistically significant, our findings have practical implication: when learning material is sufficient and provided to advanced learners in an isolated setting, one should renounce instructional prompts to save learners' time and cognitive resources.

In summary, our study's results indicate that all participants (regardless of their experimental condition) engaged in essential learning processes. The learning material's design minimized other cognitive demands (i.e., incidental processing and representational holding) in all conditions, avoiding a cognitive overload for participants. In addition, all participants could learn uninterruptedly under supervision in a university laboratory without distractions. Under these circumstances, being prompted to write down the most important working steps, to create a list of these steps in mind, to mentally rehearse the shown working steps or not being prompted at all did not result in differences in declarative knowledge about working steps (i.e., freely recalling the presented working steps in an immediate and delayed posttest) between the different conditions.

## 6 Limitations

The first limitation of our study is our posttest measure. As described above, we found no statistically significant differences in knowledge about working steps in posttests between our experimental conditions. However, our posttest measures were limited to declarative knowledge (i.e., a free recall test of the working steps displayed in the videos). One of our prompts was to give participants the instruction to mentally rehearse the working steps shown in the videos. Such mental rehearsal prompts are supposed to bridge the gap between declarative knowledge (knowing which working steps need to be executed) and procedural knowledge (knowing how these working steps should be executed correctly). As the ACT^*^ theory (Anderson, 1982) suggests, declarative knowledge is the starting point for novices on their way to acquiring a skill and becoming an expert in executing a certain task. Several studies have shown that mental rehearsal helps learners gain procedural knowledge and acquire a skill (Toth et al., 2020 for an overview). Our focus in this study was to examine in how far mental rehearsal is beneficial for gaining declarative knowledge in comparison to a control condition without instructional support. We did not test for procedural knowledge, and cannot draw any conclusions on potential differences in procedural knowledge between our experimental conditions. As our mental rehearsal condition was the only condition that prompted learners to think about how a working step was executed, it is possible that they gained more procedural knowledge than the other conditions. If mental rehearsal prompts were similarly beneficial for declarative knowledge as other prompt types (e.g., written focus prompts) and also foster procedural knowledge, this would reflect the practical implication to favor mental rehearsal prompts as instruction. Future studies should assess procedural knowledge in addition to declarative knowledge, for example by testing participants on a machine. In addition, our study should also be replicated in a setting with higher ecological validity, for example on a sample of apprentices inside a workshop.

Our study's power is another limitation. We conducted an a priori power analysis and expected medium to large effect sizes based on previous studies (e.g., Schriek et al., 2025). The estimated sample size was 128, our sample included only 117 participants in the immediate posttest measures and 94 participants in the delayed posttest. We did not attain the estimated sample size. Given the highly controlled environment and careful design of our learning material, it is possible, that the instructional prompts we used have only small beneficial effects in comparison to a control condition without prompts. However, our sample size is not large enough to detect smaller effect sizes.

Another limitation lies in how we assessed learning engagement. We relied on the *post-hoc* self-assessments participants made after having watched the tutorials and reacted to our prompts. It is important to note that such self-assessments have inherent limitations. They can be influenced by both recall errors and social desirability (e.g., Lam and Bengo, 2003). When self-assessing their learning engagement, participants might have overestimated their engagement in retrospect due to memory decay (i.e., remembering their engagement incorrectly). They might also have made wrong assessments deliberately because they thought they were supposed to appear engaged (i.e., trying to manage their impression). Additionally, the internal consistency of the learning engagement scale was α = 0.57. Schmitt (1996) argues that even lower alpha levels can be acceptable, in particular if a scale includes several content areas. This scale's validity has also been shown in other studies through high correlations between the scale and learning outcomes (e.g., Hefter, 2025; Hefter and Nitsch, 2024). However, the null findings for learning engagement might partially go back to measurement errors of the learning engagement scale, and results regarding learning engagement should be treated with caution. To further validate self-assessed learning engagement, future studies could implement behavioral assessments such as think-aloud protocols (e.g., Chi et al., 1989), eye-tracking (e.g., Lai et al., 2013) or EEG (Pi et al., 2021; Yu et al., 2022) as our study was limited to answers to prompts in a single condition as measure for cognitive engagement.

Furthermore, we cannot be certain whether or not our participants really followed the instructions given via our prompts. The prompt score indicates that participants indeed focused on working steps and wrote them in the text field. However, we only have a prompt score for the written focus condition, not for the mental focus condition and the mental rehearsal condition. Furthermore, the participants did not have the chance to go back to the video tutorials once they had received a prompt. Previous studies that worked with instructional videos paired with pop-up questions have shown that even in the absence of statistically significant differences in posttest scores, such prompts influence participants' learning behavior (Dogru et al., 2025). In their study, Dogru et al. showed that learners who received pop-up questions were more likely to rewind and rewatch the videos. Receiving a prompt to create a list of the working steps (in written form or mentally) or to mentally rehearse the working steps shown might have triggered participants to make a judgement of learning and monitor their current learning progress (e.g., Bjork et al., 2013). In other words, while reacting to our prompts, participants could have noticed that they had not fully grasped the video contents yet, and having had the chance to rewatch the video at that point could have been beneficial for learning outcomes. Such a judgement of learning could also encourage learners to ask questions about contents they did not understand. Research has shown the beneficial effect of computer-based lessons in presence of a teacher and the possibility to ask questions (Dogru and Özsevgeç, 2023). In our study, participants were supervised by student research assistants who could help participants with technical issues but who were not supposed to help in terms of content-related questions. Future studies should test the this study's prompts with the possibility for participants to rewatch the videos and assess learning analytics data (e.g., if and how often videos were paused or rewound) to see how such prompts might also influence participants' learning behavior. In addition, the presence of supervisors with content-knowledge, and the opportunity to ask content-related questions could give further insights into how such prompts change participants' learning behavior.

Lastly, our conclusions regarding cognitive load are limited to cognitive load in general. We assessed our participants cognitive load via a single open question (i.e., “How difficult did you find it to understand the content shown?”). While this approach is widely used (Brünken et al., 2010; De Jong, 2010) and enables global deductions of cognitive load, it does not differentiate between the different types of cognitive load postulated by the CLT, intrinsic load, extraneous load, and germane load. We observed no differences in (global) cognitive load between the different conditions in our study. As germane load is related to cognitive processes that are learning-relevant (e.g., facilitating the construction of relevant schemata), it should be related to parts of the learning engagement scale (e.g., via the items “I made a lot of effort to understand everything” or “I dealt with the learning content intensely”). The learning engagement scale also includes aspects of elaboration and implementation and is a measure of processing quality rather than cognitive load and the potential costs of learning (Daumiller et al., 2020; Hoffmann and Schraw, 2010). Accordingly, there is no significant correlation between learning engagement and cognitive load in our study. However, it is possible that the prompts used in this study induced cognitive load beyond germane load, for example higher extraneous load in the control condition, which did not receive any instructions to focus on certain parts of the video tutorials. Future studies should include differentiated load scales (e.g., Hart and Staveland, 1988) or secondary task measures (e.g., Korbach et al., 2018) to allow for further conclusions on how of different prompts, learning outcomes and cognitive load interrelate.

## Data Availability

The raw data supporting the conclusions of this article will be made available by the corresponding author, without undue reservation.
